# CHOREOGRAPHING INTERACTION WITHIN THE U-M HOMELAB: CONSIDERATIONS FOR AN OLDER ADULT POPULATION

**DOI:** 10.1093/geroni/igz038.3487

**Published:** 2019-11-08

**Authors:** Natalie M Leonard, Marianthie Wank, Karen Nielsen, Alicia G Carmichael, Maren Wisniewski, Vineet Raichur, Richard Gonzalez

**Affiliations:** 1 University of Michigan, Ann Arbor, Michigan, United States; 2 Georgia State University, Atlanta, Georgia, United States

## Abstract

Drawing from the lens of architecture, designing a research study for a controlled, simulated environment requires the consideration of three primary types of interaction involving people and infrastructure. These three types include the interface between and among the respondent(s) and the researcher(s), the interface between and among the people playing these roles and the infrastructure surrounding them (inclusive of self-report measures, sensing technology, furniture, etc.), and the interface between and among the various types of study infrastructure. The flow of a study across these interfaces becomes a form of choreography, with implications for protocol adherence, reproducibility, and data quality. Recently, a pilot study assessing an older adult population’s upper-body performance was our first iteration of research utilizing a simulated environment: the U-M HomeLab, an ADA-accommodating, one-bedroom apartment built within the basement of a large research facility. Nine participants, aged 61 to 72, with self-reported upper-body weakness completed a series of tasks resembling activities of daily living, such as lifting laundry baskets and vacuuming. By backtracking through our development of this pilot study, we illustrate how considerations of interface play a significant role across every stage of study design, incorporating aspects of wayfinding, dialogue, safety, acclimation, and visibility that are relevant to an older adult population. From these reflections, considerations of interface inform a “check-list” for simulation choreography, providing guiding questions for assessing these types of interactions while iterating through study design.

